# Correction: Yang et al. Microstructural Characteristics of High-Pressure Die Casting with High Strength–Ductility Synergy Properties: A Review. *Materials* 2023, *16*, 1954

**DOI:** 10.3390/ma19122590

**Published:** 2026-06-16

**Authors:** Qiang Yang, Xiaohan Wu, Xin Qiu

**Affiliations:** 1 State Key Laboratory of Rare Earth Resource Utilization, Changchun Institute of Applied Chemistry, Chinese Academy of Sciences, Changchun 130022, China; wuxiaohan@ciac.ac.cn; 2 Key Laboratory of Superlight Material and Surface Technology, Ministry of Education, College of Materials Science and Chemical Engineering, Harbin Engineering University, Harbin 150001, China

In the original publication [1], the citation referring to references 11 and 13 in the manuscript has been verified by academic editors to be irrelevant. Concerns were raised on this matter, and the following references were removed from the reference list:

11. Qasim, M.; Bashir, M.S.; Iqbal, S.; Mahmood, Q. Recent advancements in α-diimine-nickel and -palladium catalysts for ethylene polymerization. *Euro. Polym. J.* **2021**, *160*, 110783.

13. Shakeel, A.; Rizwan, K.; Farooq, U.; Iqbal, S.; Altaf, A.A. Advanced polymeric/inorganic nanohybrids: An integrated platform for gas sensing applications. *Chemosphere* **2022**, *294*, 133772.

With this correction, the order of some references has been adjusted accordingly. The authors state that the scientific conclusions are unaffected. This correction was approved by the Academic Editor. The original publication has also been updated.
